# Effects of different harvesting methods on soil bacterial diversity in *Betula platyphylla* secondary forest in Daxing 'an Mountains, inner Mongolia

**DOI:** 10.3389/fmicb.2026.1716131

**Published:** 2026-04-13

**Authors:** Kaitao Zhai, Shengwei Liu, Hao Zhang, Rula Sa

**Affiliations:** 1 College of Forestry, Inner Mongolia Agricultural University, Hohhot, China; 2 National Field Scientific Observation and Research Station of Greater Khingan Forest Ecosystem, Genhe, China

**Keywords:** bacterial diversity, *Betula platyphylla* secondary forests, Daxing 'an Mountains, logging methods, soil physical and chemical properties

## Abstract

**Introduction:**

In the context of global climate change, optimizing forest management is essential for improving cold-temperate forest ecosystems. However, the pathways through which forest management influences ecological functions via soil and microbial communities remain unclear.

**Methods:**

This study examined how logging methods impact the soil properties and bacterial communities in the Daxing 'an Mountains, Inner Mongolia. Sample plots for five conditions were analyzed: secondary gradual cutting (S1 and S2), secondary gradual cutting with replanting (S3 and S4), clear cutting (ML and MB), and original forests (L1, L2, and L3).

**Results:**

Logging methods drove distinct soil ecosystem responses among plots, mediated by long-term disturbance legacies and successional processes. (1) ML had the highest pH, total nitrogen, total phosphorus, and capillary porosity. MB showed a favorable nutrient status, characterized by high total potassium. S1 and S4 demonstrated good organic matter and physical structure, while S2 and S3 showed nutrient imbalances or structural issues. Notably, L1-L3 displayed the poorest nutrient levels and physical properties. (2) All plots shared Proteobacteria, Acidobacteriota, and Actinobacteriota as core phyla, with specific enrichments: Verrucomicrobiota in ML; Chloroflexi in S3-S4; and Planctomycetota in L1-L3. Diversity was highest in L1 and lowest in L3, with high levels in ML, MB, and S3 and lower levels in S1, S2, and S4. Community composition was similar among S4, ML, and MB but distinct in other plots. Bacterial biomarkers were significantly associated with soil factors (p < 0.05): A21b_unclassified (S1), *Granulicella* (S2), Gemmataceae_unclassified (S3), AD3_unclassified (S4), KD4-96_unclassified (ML), RB41 (MB), and Vicinamibacterales_unclassified (L1). PICRUSt2 predictions may indicate potential increases in metabolism (S2, L1, and L3), environmental information processing (S3, L1, and L3), genetic information processing (S4 and ML), and cellular processes (MB and L3). (3) Soil factor analysis identified pH as the original community driver overall. Total nitrogen and soil organic matter dominated in cutting plots, while moisture content was key in forest plots.

**Conclusions:**

This study found that appropriate logging methods improve soil nutrient content and bacterial diversity by regulating soil pH, total nitrogen, and moisture. These results demonstrate the profound impact of forest management on core soil ecological processes.

## 1 Introduction

Bacteria play a dominant role in the functioning of soil ecosystems and drive the cycling of nutrients and energy in forest ecosystems (Delmont et al., 2012; Li et al., 2024). Their community structure and function directly affect soil nutrient availability, carbon and nitrogen cycling, and vegetation restoration efficiency. Changes in bacterial diversity indicate soil health and help guide forest restoration strategies (Lu et al., 2013). The composition of soil bacteria is also a critical factor in determining soil fertility and ecosystem restoration in degraded areas (Peng, 2018; Zhang, 2023).

The Daxing 'an Mountains region has experienced prolonged and intensive logging, which has resulted in widespread conversion of original forests to secondary and plantation forests. This transformation has substantially altered soil physicochemical properties and driven compositional and functional shifts in soil bacterial communities (Wang et al., 2019). Different logging methods differentially alter canopy structure, reduce litter input, and change microenvironmental conditions. Canopy thinning allows sunlight to penetrate, leading to significant loss of nutrients and increased soil erosion, which further drives the succession and functional reorganization of bacterial communities (Zhang S. et al., 2023; Ahn et al., 2012). Various logging methods impact the physical and chemical properties of the soil differently. For example, clear cutting, a high-intensity logging method, removes a large amount of vegetation, resulting in a significant loss of organic matter in the soil surface layer, destruction of soil structure, reduced soil water-holding capacity, accelerated loss of available nitrogen, phosphorus and other nutrients, and increased pH (Liu, 2014; Zhang X. et al., 2023). In contrast, low-intensity logging methods such as gradual cutting do not significantly damage the soil structure and nutrient cycling because they retain part of the canopy (Teng et al., 2023). This logging method maintains litter input and microclimate stability and results in only a small loss of organic matter.

Research has shown that logging intensity affects soil bacterial diversity in complex ways. For example, clear-cutting significantly reduces soil bacterial alpha diversity, likely due to the loss of organic matter, while gradual cutting supports bacterial recovery. For example, studies in the clear-cutting plots of the Daxing 'an Mountains have reported that the abundance of oligotrophic bacteria (e.g., Acidobacteria) decreases as organic matter content declines, whereas copiotrophic bacteria (e.g., Proteobacteria) initially increase and then decrease due to fluctuations in available carbon sources (Li et al., 2019). In contrast, moderately gradual cutting helps maintain the litter input and microenvironmental stability, and it promotes the recovery of functional bacterial groups such as *Nitrospirae*, which are involved in the nitrogen cycle (Zhang, 2024). Additionally, soil compaction resulting from logging reduces soil porosity, which can inhibit the growth of obligate aerobes (strict aerobic bacteria such as *Actinobacteria*), further affecting the efficiency of organic matter decomposition (Fu et al., 2025). The physical and chemical properties of soil are the core factors driving the differentiation of the bacterial community structure and function. For example, soil pH is the dominant factor affecting bacterial distribution, with Acidobacteria thriving in acidic soils and Proteobacteria flourishing in neutral soils (Cai et al., 2024). Studies have also shown that total phosphorus is significantly and positively correlated with *Actinobacteria*, whereas available nitrogen affects carbon cycling efficiency by inhibiting Chloroflexi (Cheng, 2017). Moreover, organic matter and soil moisture content significantly affect competition between aerobic bacteria (e.g., Proteobacteria) and anaerobic bacteria (e.g., Firmicutes) by regulating the rate of oxygen diffusion (Sun et al., 2018).

The Daxing ‘an Mountains forest area, an important component of boreal coniferous forests in northern Eurasia, plays an irreplaceable role in carbon sequestration and water conservation (Liu et al., 2024). However, since the mid-20th century, large-scale logging has led to rapid degradation of the original mixed coniferous and broad-leaved forests, replacing them with secondary forests dominated by *Betula platyphylla*. These secondary birch forests represent a key transitional stage of forest succession. Soil bacterial diversity in these forests is crucial for maintaining carbon and nitrogen cycles, organic matter decomposition and ecosystem restoration (Wang Z. et al., 2024). A fundamental question that remains unanswered in the context of cold-temperate *Betula platyphylla* secondary forests is: what mechanisms govern the interactions among logging intensity, soil properties, and bacterial diversity across different successional stages? This study was therefore designed to systematically clarify these complex relationships and the underlying regulatory pathways involving ecological stoichiometry and functional genes. Therefore, this study focused on birch secondary forest in the Daxing'an Mountains and aimed to reveal (1) influence of different logging methods on soil physical and chemical properties; (2) the influence of different logging methods on soil bacterial communities, identify key biomarkers in each plot, and predict the functional composition of different plots; and (3) the correlation characteristics between soil physical and chemical properties and bacterial communities. The results may provide valuable insights for optimizing the “near-natural management” strategy for cold-temperate forests and preserving ecological barriers against the background of global climate change.

## 2 Materials and methods

### 2.1 Overview of the research site

**Figure 1:**
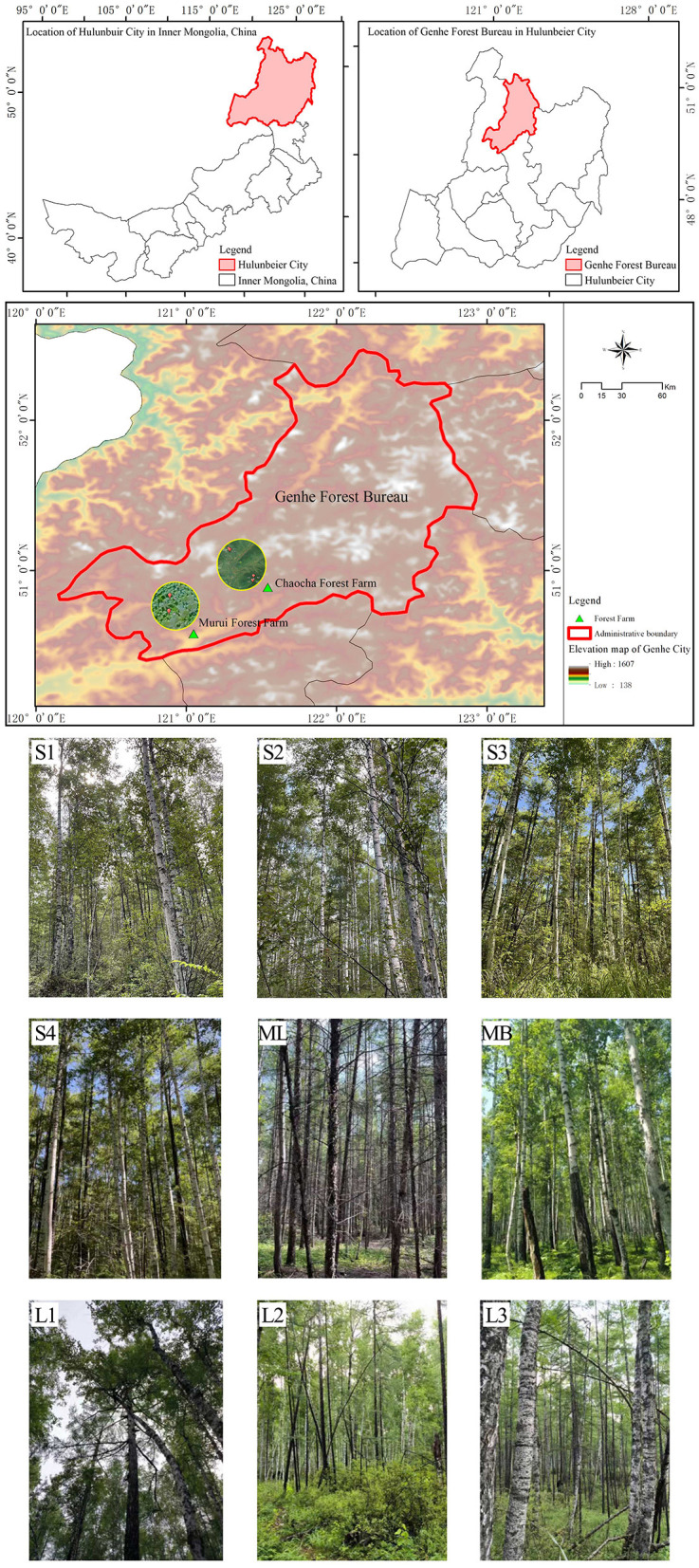
Map of the study area.

The study area is located in the northern forested area of the Daxing'an Mountains, Inner Mongolia, China. In 2020, secondary gradual cutting of plots S1 and S2 (harvested in 1990) and secondary gradual cutting and replanting of plots S3 and S4 (harvested in 1990) were completed at the Chaocha Forest Farm of the Genhe Forest Bureau; in addition, original forest plots L1, L2, and L3 (80-year stand age) were also included in this study. Further, clear-cutting of plot ML and MB (harvested in 1994) were completed at Murui Forest Farm. Plots L1–L3 were situated within zonal climax old-growth stands representing late-successional stages free from intensive anthropogenic disturbance. These stands served as the reference ecosystem for this study, representing the most natural and stable forest conditions in the local landscape. Six 30 × 30-m sampling plots were set up, and retests were conducted in 2022 (Table 1). The location and scope of the study area are shown in Figure 1. The study area is mainly composed of gentle mountains with an altitude of 700–1530 m, an average annual temperature of−5.3 °C and an average annual precipitation of 500 mm. These sites comprise predominantly brown coniferous forest-type soil, which is acidic and contains more sand and gravel. The study area is rich in species resources and its forests are dominated by *Larix gmelina* and *Betula platyphylla* The main shrubs in the forest included *Ledum palustre, Rhododendron simsii Planch, Carex tristachya Thunb*, and *Melica virgata Turcz*.

**Table 1:** Sample plot information table.

Jamming mode	Plot name	Altitude (m)	Aspect of slope	Canopy density	Mixing degree	Mean diameter (cm)	Average tree height (m)	Tree species composition	Shrub richness	Herb richness
Secondary gradual cutting disturbance	Gradual cutting plot S1	969	Southwest slope	0.76	0.07	11.26	12.78	Birch pure forest	11	6
	Gradual cutting plot S2	973	Western slope	0.64	0.15	17.43	16.67	9 white 1 fall	8	3
	Gradual cutting and replanting plot S3	740	Southwest slope	0.64	0.08	9.26	11.14	8 white 2 fall	10	6
	Gradual cutting and replanting plot S4	736	Southwest slope	0.60	0.25	9.80	10.02	6 fall 4 white	8	9
Clear cutting interference	Artificially planted plot ML	710	East slope	0.60	0.28	9.79	14.55	9 fall 1 white	4	7
	Natural birch forest plot MB	705	East slope	0.60	0.15	16.17	16.00	9 white 1 mountain poplar	5	12
Original forest	Original forest plot L1	877	Northwest slope	0.62	0.26	11.79	14.48	7 fall 3 white	8	13
	Original forest plot L2	877	Northwest slope	0.64	0.28	15.31	14.60	6 fall 4 white	7	9
	Original forest plot L3	877	Northwest slope	0.60	0.24	11.77	10.72	7 fall 3 white	5	8

### 2.2 Soil sample collection

Three quadrats were evenly arranged along a diagonal of each sample plot. The hummus layer on the soil surface was removed before sampling. Samples from the 0-20 cm soil layer were collected using a soil auger and a ring knife. All sub-samples from the same plot were mixed evenly. The soil samples were passed through a 2-mm sieve to remove stones, litter, and other debris and then divided and numbered. The soil samples were stored in 10-mL centrifuge tubes at−20 °C for soil bacteria measurements and in sterile sealed bags at 4 °C for soil physicochemical property measurements and then dried in time with a ring knife. The names and numbers of the trees, shrubs and herbs in each plot were also recorded during soil sample collection.

### 2.3 Measurement of soil physical and chemical properties

In this study, the pH, soil organic matter (SOM), soil organic carbon (SOC), total nitrogen (TN), total phosphorus (TP), total potassium (TK), moisture (MC) contents, and bulk density (BD), capillary porosity (CP), and non-capillary porosity (NCP) of the soil were measured. The methods of determining the physical and chemical properties are provided in Table 2.

**Table 2:** Soil determination method table.

Measurement index	Determination method	Instrument information
pH value	‘Soil testing - Part 2: Determination of Soil pH' (Ministry of Agriculture of the People's Republic of China, 2006a)	ST3100 pH meter (NRJJ-S-020)
Organic matter content	‘Soil testing-Part 6 : Determination of soil organic matter' (Ministry of Agriculture of the People's Republic of China, 2006b)	Acid brown (50 ml) burette (NRJJ-S-193 1)
Organic carbon content	‘Determination of soil organic carbon ‘Potassium dichromate oxidation-spectrophotometric method' (Ministry of Environmental Protection of the People's Republic of China, 2011)	UV-1800 UV-visible spectrophotometer (NRJJ-S-031)
Total nitrogen content	‘Soil testing–Part 24: Determination of soil total nitrogen–Automatic nitrogen analyzer method' (Ministry of Agriculture of the People's Republic of China, 2012)	K1100 Automatic Kjeldahl Nitrogen Analyzer (NRJJ-S-013)
Total phosphorus content	‘Soil total phosphorus determination method' (Ministry of Agriculture of the People's Republic of China, 1988)	UV-1800 UV-visible spectrophotometer (NRJJ-S-031)
Total potassium content	‘Determination of potassium in forest soil' (3 Determination of total potassium) (State Forestry Administration., 2015)	FP6410 Flame Photometer (NRJJ-S-294)
Soil moisture content	Drying and weighing method (Zhang and Gong, 2012)	Ring knife (volume 100 cm3)
Soil bulk density	Ring knife method (Zhang and Gong, 2012)	
Soil capillary porosity	Ring knife saturated water absorption method (Zhang and Gong, 2012)	
Soil non-capillary porosity		

Soil capillary porosity and soil non-capillary porosity were both determined using the ring knife saturated water absorption method (Zhang and Gong, 2012). A 100 cm^3^ ring knife was used as the measuring instrument for soil moisture content, soil bulk density, soil capillary porosity and soil non-capillary porosity.

### 2.4 High throughput sequencing of soil bacteria

The cetyltri methyl ammonium bromide (CTAB) method was used to extract the total DNA of microorganisms in soil samples, the quality of DNA extraction was assessed using agarose gel electrophoresis and the DNA was quantified using an ultraviolet spectrophotometer. Polymerase chain reaction (PCR) amplification of the samples was performed using 50 mg of template DNA. The thermal cycle conditions for PCR amplification were as follows: initial denaturation at 98 °C for 30 s; then 32 cycles of 98 °C for 10 s, 54 °C for 30 s, and 72 °C for 45 s; and a final lengthening cycle at 72 °C for 10 min. The primers used were 515F 5′-GTGYCAGCMGCCGCGGTAA3′) and 806R 5′-GGACTACHVGGGTWTCTAAT3′) for PCR amplification of the 16S rDNA variable region 4 (V4). PCR products were purified using AMPure XT beads (Beckman Coulter Genomics, Danvers, MA, USA) and quantified using a Qubit (Invitrogen, Waltham, MA, USA). PCR products were detected using 2% agarose gel electrophoresis and recovered using an AMPure XT bead recovery kit. The purified PCR products were evaluated using an Agilent 2100 Bioanalyzer (Agilent, Santa Clara, CA, USA) and library quantification kits (Kapa Biosciences, Woburn, MA, USA). The concentration of the qualified library was greater than 2 nM. After gradient dilution of the qualified sequencing libraries (index sequence was not repeatable), they were mixed in the corresponding proportion according to the required sequencing amount and denatured using NaOH to a single strand for sequencing. A NovaSeq 6000 sequencing instrument was used for 2 × 250 bp double-ended sequencing using the corresponding reagents supplied in the NovaSeq 6000 SP reagent kit (500 cycles).

For the double-ended data obtained by sequencing, the samples were split according to barcode information and the joints and barcode sequences were removed. Cut adapt software was used to remove the primer sequence and the balanced base sequences of the raw data. Each pair of paired-end reads was spliced into a longer tag according to the overlapping region using FLASH software. The Fqtrim software was used to scan the quality of the sequencing reads using the window method. The default scanning window was 100 bps. When the average mass value in the window was less than 20, the part of read from the beginning of the window to the 3′ end was cut off. The sequences with lengths less than 100 bp and the sequences with uncertain fuzzy base content of greater than 5% were removed. Chimeric sequences were removed using V search software. Using the QIIME DADA2 software program paired-end reads were de noised and length filtering was also performed. Amplicon sequence variant (ASV) feature sequences and an ASV abundance table were obtained, and singleton ASVs (i.e., ASVs with a total number of sequences of only one in all samples) were removed.

### 2.5 Statistical analysis

The soil data were tested for normality (Shapiro-Wilk test) and homogeneity of variance (Bartlett test) using R version 4.1.3. Single-factor analysis of variance (ANOVA) was performed on the soil data. Based on the ANOVA results, multiple comparisons and pound-to-pair comparisons were conducted and Tukey's HSD test (significance level was 0.05 by default) was used to explore the differences among samples. Based on the ASV (feature) sequence file, species annotation was performed using the SILVA and NT-16S databases and the abundance of each species in each sample was counted according to the ASV (feature) abundance table. The confidence threshold for the comments was 0.7.

Alpha and beta diversity analyses were performed based on the obtained ASV (feature) sequence and ASV (feature) abundance table. Alpha diversity refers to the diversity within a specific environment or ecosystem and was used to analyse the microbial community diversity within a sample, reflecting the richness and diversity of the microbial community in different places. In this study, ASVs and the Shannon, Simpson, and Chao1 indices were used to reflect differences in microbial richness and diversity. Beta diversity refers to species diversity between different environmental communities. Beta and alpha diversity constitute the overall diversity or biological heterogeneity of a given environmental community. In this study, non-multidimensional scaling analysis (NMDS) and multivariate analysis of variance (permutational multivariate analysis of variance (PERMANOVA), also known as Adonis) were used to observe the differences in microorganisms in different locations.

Indicator analysis is a widely used classical amplicon data analysis method that can identify biomarkers with significant indicative values. These biomarkers play a key role in effectively indicating sample groupings and other characteristics. Phylogenetic Investigation of Communities by Reconstruction of Unobserved States (PICRUSt) is a tool for predicting functional abundance based on marker gene sequences (Langille et al., 2013). It can predict functions such as gene families (e.g., KEGG orthologs) and any arbitrary traits, with 16S rDNA data as the common input, and it also supports the predictions of other marker genes.

R version 4.1.3 was used to draw the following: box plots of soil physicochemical properties and bacterial diversity (ggplot2 package); Circos plots of the relative abundance of bacteria at the phylum level (circlize package); histograms of the percentage of the relative abundance of bacteria at the genus level (ggplot2 package); Sankey diagram of soil bacterial phylum-genus level relative abundance associations (ggalluvial package); bacterial NMDS map (ggplot2 and vegan packages); correlation network map (igraph package); soil bacterial indicator analysis bubble chart (ggplot2 and omicstudioclassic package); and soil bacterial functional prediction STAMP difference analysis map (ggplot2 and omicstudiokits package).

All genomic analyses were performed according to the manufacturer's instructions. All trials were repeated thrice, and the data were analyzed using ANOVA and Duncan's multiple range test. Differences with *p* values < 0.05 were considered statistically significant. All sampling plots served as spatially independent replicates with consistent topography, soil parent material, and stand disturbance history. Due to the scarcity of well-preserved forest sites exhibiting undisturbed logging histories in the study region, replication was restricted to two plots for most logging treatments and three for original forests.

## 3 Results and analysis

### 3.1 Soil properties analysis

Acomparative analysis of the plots revealed generally high soil nutrient content and favorable physical properties. Specifically, soil nutrient content (SOM, SOC, TN, and TK) and physical properties (MC and CP) were high with considerable fluctuations; BD was high with insignificant fluctuation; TP and NCP were low with insignificant fluctuation; and pH was weakly acidic with insignificant fluctuation. Compared with the original forest plot, the cutting plot exhibited increases in key soil properties: soil nutrients (pH, SOM, SOC, TN, TP, and TK) increased by 7.79%, 151.79%, 217.56%, 26.32%, 60.61%, and 26.34%, respectively; physical properties (MC, CP, and NCP) increased by 28.32%, 3.57%, and 79.21%, respectively. In contrast, BD decreased by 20.18% (Table 3).

**Table 3:** Descriptive statistics of soil indicators.

Soil physical and chemical characteristics	Cutting plot				Original forest plot			
	Mean value	Standard deviation	Minimum value	Maximum value	Mean value	Standard deviation	Minimum value	Maximum value
pH value	5.26	0.53	4.66	6.00	4.88	0.27	4.57	5.31
Organic matter content (g/kg)	135.64	47.27	72.40	245.50	53.87	20.48	26.60	89.15
Organic carbon content (g/kg)	8.86	3.07	4.73	15.94	2.79	1.06	1.48	4.71
Total nitrogen content (g/kg)	3.84	1.88	1.47	7.61	3.04	1.33	1.43	5.23
Total phosphorus content (g/kg)	1.06	0.48	0.45	1.90	0.66	0.17	0.39	0.93
Total potassium content (g/kg)	20.58	1.19	18.05	22.05	16.29	1.50	13.05	17.90
Soil bulk density (g/cm^3^)	0.91	0.12	0.65	1.12	1.14	0.19	0.85	1.40
Soil moisture content (%)	28.68	4.18	21.33	38.02	22.35	3.47	15.68	27.33
Soil capillary porosity(%)	58.53	7.17	48.94	74.66	56.51	8.97	45.20	71.11
Soil non-capillary porosity (%)	3.19	1.27	1.11	4.95	1.78	0.34	1.10	2.29

**Figure 2:**
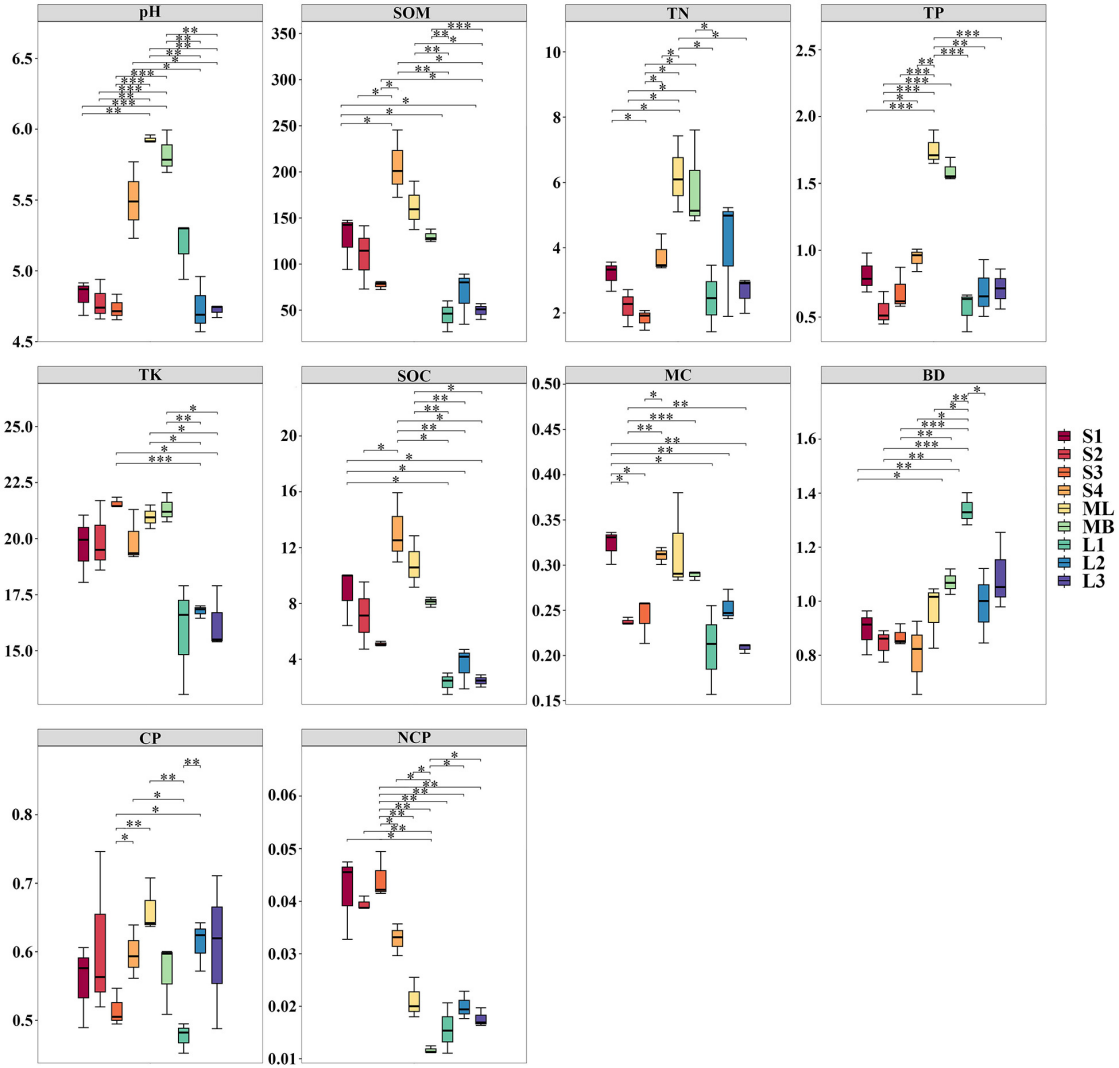
Boxplots of the soil physical and chemical properties of the *Betula platyphylla* secondary forest under different harvesting methods. S1 and S2 are gradual cutting plots, S3 and S4 are gradual cutting and replanting plots, ML is a clear cutting plot, MB is a natural birch forest plot and L1, L2, and L3 are original forest plots. The upper quartile represents the 75th percentile (Q3) of the data, and the lower quartile represents the 25th percentile (Q1). The height of the box (Q3–Q1) reflects the data dispersion (middle 50%), with the smaller boxes indicating more concentrated data. The thick line inside the box represents the median (or 50th percentile). The whiskers represent the range of data, with its length determined by the maximum and minimum values of the data. * Significant differences among different plots (p < 0.05), ** very significant differences among different plots (p < 0.01) and *** extremely significant differences among different plots (p < 0.001). Soil organic matter (SOM), total nitrogen (TN), total phosphorus (TP), total potassium (TK), soil organic carbon (SOC), water (MC), bulk density (BD), capillary porosity (CP), and non-capillary porosity (NCP).

Soil chemical and physical properties varied considerably across the different sample plots (Figure 2). Regarding chemical properties, a distinct nutrient gradient was observed. Plot ML demonstrated comprehensive nutrient advantages, with the highest soil pH and the highest TN and TP contents among all plots, differing significantly from most others (*p* < 0.05 to *p* < 0.001). It also exhibited high SOM and SOC. Plot MB maintained good soil nutrient content, with pH, TN, and TP second only to ML. Moreover, MB had a high TK content, even exceeding that of ML, although its SOM and SOC were only moderate. Plot S1 had relatively high SOM and SOC; S2 showed lower TN and the lowest TP; S3 displayed severe nutrient imbalance, with the lowest TN but the highest TK; and S4 had the highest SOM and SOC contents overall. Plot L1 had the lowest SOM, L2 the lowest pH, and L3 the lowest TK and SOC among all plots.

In terms of physical properties, different treatments influenced soil structure. For example, plot ML displayed the best pore characteristics, with the highest CP (significantly higher than that in S3, p < 0.01), which indicated excellent water-holding capacity, although its NCP was relatively low. Plot MB displayed generally favorable physical properties, with high MC and moderate BD, but it had the lowest NCP among all plots; this pattern suggested potential limitations in aeration and permeability. Plot S1 had an excellent physical structure, with the highest MC and NCP, indicating good water retention and aeration. S2 represented the opposite extreme, with the lowest BD but also low NCP, resulting in loose soil. S3 performed poorly overall, whereas S4 appeared relatively favorable, with MC and CP in the upper-middle range. Plot L1 had the lowest BD, L3 had the lowest MC, and all three L-plots showed generally low CP and NCP; these characteristics collectively indicated deteriorated soil physical structure.

### 3.2 Soil bacterial abundance analysis

**Figure 3:**
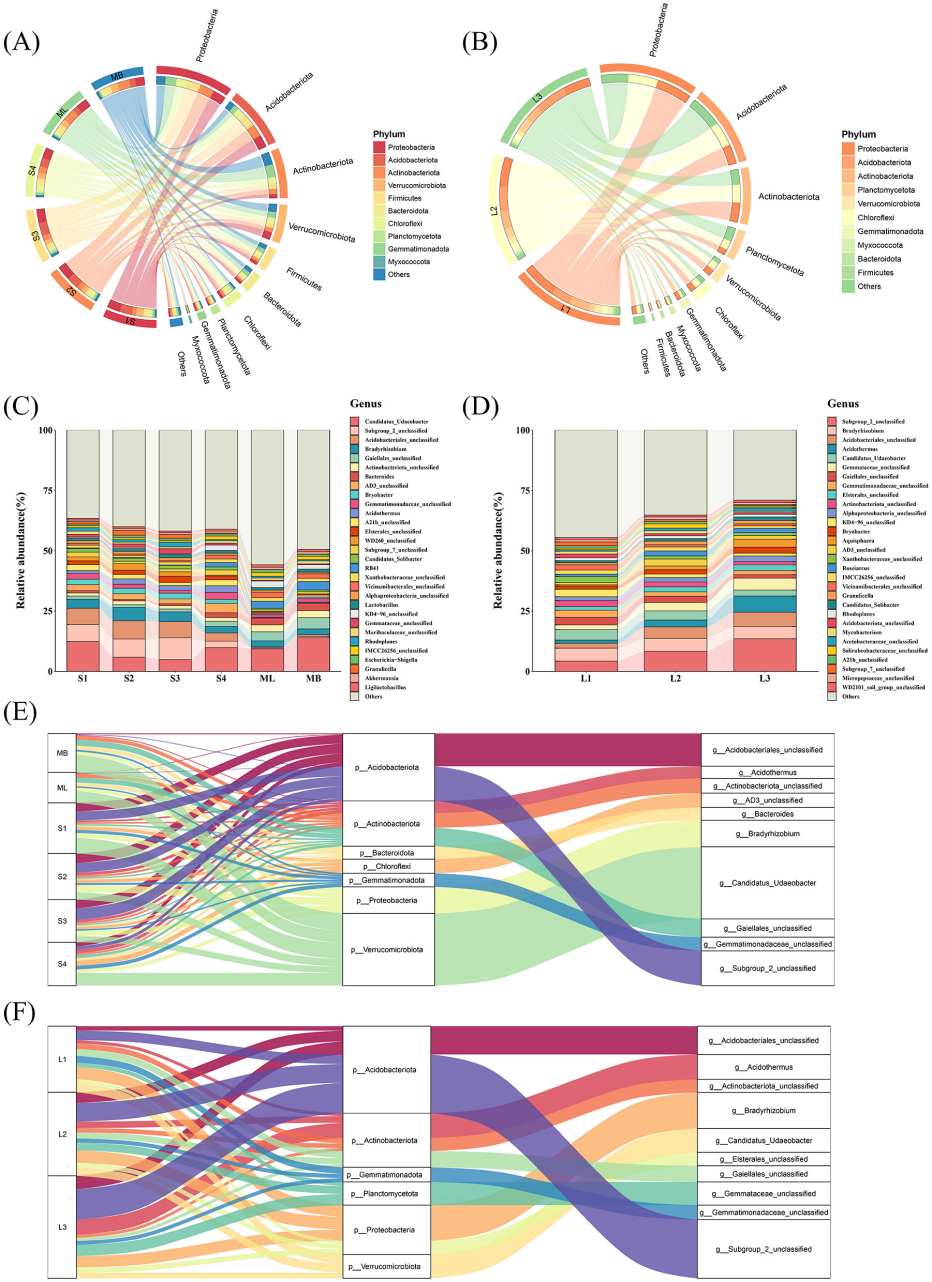
Relative abundance of soil bacteria in *Betula platyphylla* secondary forests under different harvesting methods. **(A)** and **(B)** Relative abundance of soil bacteria at the phylum level (16S rRNA gene copy number) in the cutting and original forest plots. The left half shows the top 10 phyla and their corresponding abundance data, with wider sections representing higher abundance. The right half is the grouping information displayed in accordance with the grouping. **(C)** and **(D)** Relative abundance of top 30 bacterial genera in the cutting and original forest plots. **(E)** and **(F)** Sankey diagram showing the relative abundances of the bacterial communities at the genus level **(middle)** and the species level **(right)** corresponding to different plots (on the left). The diagram visually presents the species annotation information, corresponding relationships, and proportions of phyla and genera in the study of bacterial community diversity.

Based on species annotation and abundance data, the top 10 bacterial phyla and top 30 bacterial genera were selected for analysis. At the phylum level, both cutting and original forest plots shared similar dominant phyla but showed notable abundance variations. Proteobacteria (24.04% vs 29.02%), Acidobacteriota (19.18% vs 23.89%), and Actinobacteriota (15.51% vs 16.68%) formed the core communities in both plot types. Verrucomicrobiota was more abundant in the cutting plot (11.93% vs 5.96%), whereas Planctomycetota occurred more frequently in the original forests (2.68% vs 8.98%). Chloroflexi appeared at similar proportions (5.81% vs 5.54%) (Figures 3A, B). At the genus level, the dominant genera in both plot types included *Candidatus Udaeobacter* (9.38% vs 3.54%), Subgroup_2_unclassified (4.52% vs 8.67%), and Acidobacteriales_unclassified (4.27% vs 4.20%). The cutting plots contained abundant *Bacteroides* (1.68%) and *A21b_unclassified* (1.51%), whereas original forest plots included abundant Gemmataceae_unclassified (3.42%) and Elsterales_unclassified (1.98%). The “others” category accounted for substantial proportions in both plot types (44.13% vs 36.26%) (Figures 3C, D).

Further analysis of individual plots revealed specific distribution characteristics. Plot ML contained a relatively high abundance of Proteobacteria, Actinobacteriota, *Candidatus Udaeobacter*, and *Gaiellales_unclassified*. Plot MB had contained relatively high Proteobacteria, Actinobacteriota, Verrucomicrobiota, *Candidatus Udaeobacter, Bacteroides*, and RB41. Plots S1 and S2 contained a relatively high abundance of Proteobacteria, Actinobacteria, *Candidatus Udaeobacter*, Subgroup_2_unclassified, Acidobacteriales_unclassified, and *Bradyrhizobium*. Plots S3 and S4 contained a relatively high abundance of Proteobacteria, Acidobacteriota, Actinobacteriota, Chloroflexi, Subgroup_2_unclassified, and Acidobacteriales_unclassified. Plots L1, L2, and L3 contained a relatively high abundance of Proteobacteria, Acidobacteriota, Actinobacteriota, Planctomycetota, Subgroup_2_unclassified, Acidobacteriales_unclassified, *Bradyrhizobium*, and *Candidatus Udaeobacter* (Figures 3A–D).

Sankey diagram analysis revealed distinct hierarchical distribution patterns of soil bacteria between the cutting and original forest plots at both phylum and genus levels. The cutting and original forest plots had seven and six dominant phyla, respectively, while the downstream-region plot contained 10 dominant phyla in both plot types. The strongest phylum–genus associations differed substantially: in the cutting plots, the dominant associations were Verrucomicrobiota–*Candidatus Udaeobacter* (28.22% edge width) and Acidobacteriota–Subgroup 2 unclassified/Acidobacteriales_unclassified (22.51%); in the original forest plots, the dominant associations were Acidobacteriota Subgroup 2 unclassified/Acidobacteriales_unclassified (33.16%) and Proteobacteria–*Bradyrhizobium*/Elsterales_unclassified (27.30%). Notably, the flow distribution within Acidobacteriota differed between plot types. The distribution proportion of Subgroup 2 unclassified was higher in the original forest plots (23.26%) than in the cutting plots (13.76%), while Acidobacteriales_unclassified showed similar proportions in both plot types (13.02% and 11.25%) (Figures 3E, F). These findings not only confirm that the combination of Acidobacteriota with Subgroup_2_unclassified and Acidobacteriales_unclassified forms a core bacterial community composition unit across all samples but also show that Subgroup_2_unclassified plays a dominant role in shaping community structure within this phylum.

### 3.3 Soil bacterial diversity analysis

**Figure 4:**
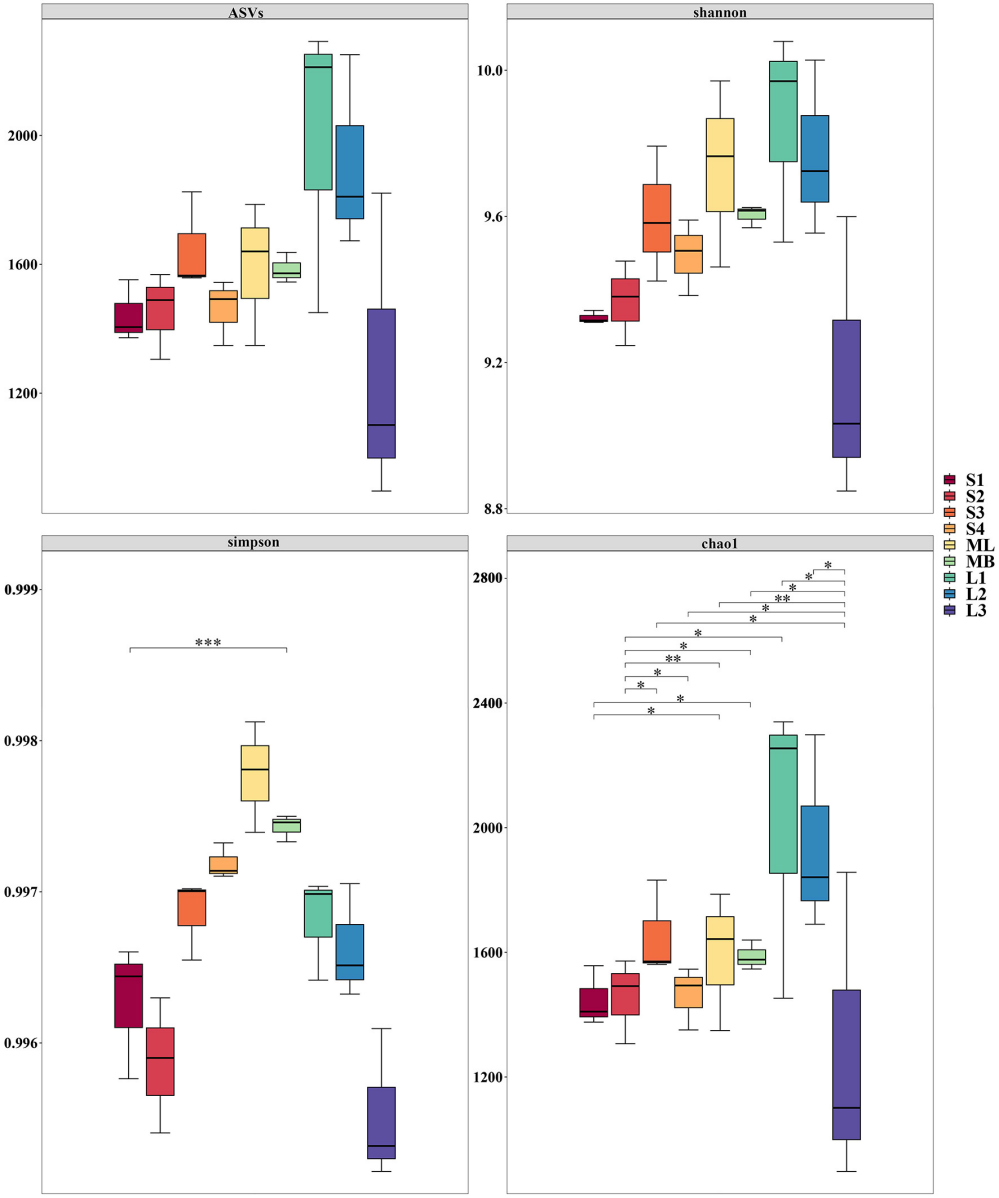
Boxplots of the alpha diversity indices for soil bacteria in *Betula platyphylla* secondary forest under different harvesting methods. Figures show the Chao1, amplicon sequence variant (ASV), Shannon, and Simpson index box plots for the cutting and original forest plots. The Chao1 index estimates the total number of species contained in the community samples and also reflects the presence of low-abundance species. The ASV represents microbial taxa based on precise nucleotide differences in sequencing data. The Shannon index indicates diversity, with higher values representing greater uncertainty and diversity. The Simpson index ranges from 0 to 1, with higher values indicating greater diversity and evenness. Statistical significance is indicated by * (*p* < 0.05), ** (*p* < 0.01), and *** (*p* < 0.001). Amplicon sequence variant (ASV).

Analysis of alpha diversity indices revealed significant differences in soil bacterial diversity between the cutting and original forest plots (Figure 4). Plot L1 consistently exhibited the highest microbial diversity, with the maximum values for ASVs, Shannon, and Chao1 indices. Similarly, L2 maintained elevated diversity across these indices, while L3 showed the lowest diversity levels among all plots. Plot ML demonstrated strong diversity performance and ranked high in multiple indices and achieved the highest Simpson index value. Plot MB displayed intermediate diversity levels, with its Simpson index significantly exceeding that of S2 (*p* < 0.001). Plots S1 and S2 generally displayed lower diversity levels, with S1 and S2 showing significantly lower Chao1 indices compared to that of plot MB (*p* < 0.05). Plots S3 and S4 demonstrated varied patterns: S3 maintained relatively high diversity across ASVs, Shannon, and Chao1 indices, while S4 showed lower diversity levels except for the Simpson index (Figure 4).

**Figure 5:**
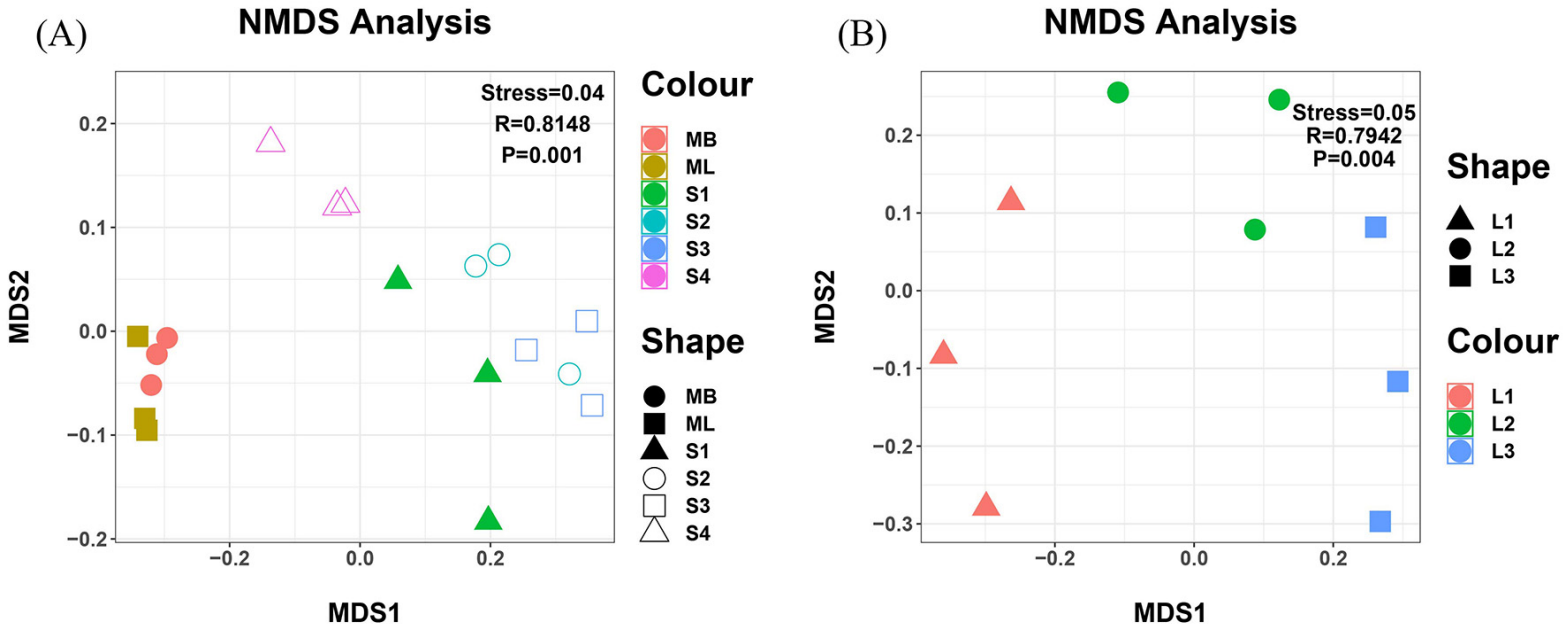
NMDS diagram of soil bacteria in *Betula platyphylla* secondary forest under different harvesting methods. **(A)** and **(B)** Bacterial NMDS maps for the cutting and original forest plots. The points represent samples, with colors indicating different groups. The distance between points the degree of difference between the samples. The R values, ranging from−1 to 1, indicates the difference between group and within group sample differences. R values closer to 1 suggest better grouping, with a smaller difference within groups. R = 0 suggests random grouping with no observable statistical difference between the sample groups. A negative R value indicates that the sample difference within the group exceeds the sample difference between the groups, leading to poor grouping. The *P* value reflects the statistical significance of the ANOSIM analysis result. The stress coefficient (stress) measures the NMDS analysis quality, with stress < 0.2 a meaningful two-dimensional representation. Stress coefficient < 0. suggests a good sort and stress coefficient < 0.05, indicates good representativeness. Non-multidimensional scaling analysis (NMDS); Analysis of similarity (ANOISM).

NMDS analysis revealed significant clustering patterns in bacterial community composition across the different plots (Figure 5). Among the cutting plots, S1, S2, and S3 formed distinct clusters separated by large distances, while S4, ML, and MB formed a close cluster, which indicated similar community structures. Similarly, within the original forest plots, L1, L2, and L3 showed substantial separation from one another. These spatial patterns reflect distinct bacterial community relationships: S1, S2, S3, L1, L2, and L3 each maintained unique community compositions with considerable differences, whereas S4, ML, and MB shared similar community characteristics with minimal variation.

PERMANOVA further confirmed these patterns and showed high R values for both cutting and original forest plots. This observation indicates strong between-group differentiation relative to within-group variation, which supports the distinct clustering observed. The statistically significant *p*-values for bacterial communities (*p* < 0.05) indicate meaningful differences in community composition between the cutting and original forest treatments. Additionally, the stress value below 0.1 validates that the NMDS ordination accurately represents the bacterial community relationships, with all samples showing clear separation in the reduced dimensional space.

### 3.4 Soil bacterial indicator analysis

Based on the bacterial community composition at the phylum and genus levels, an indicator species analysis was performed on the top 30 most abundant species to identify biomarkers across different plots, to determine key species indicative of plot grouping, and to elucidate functional differences among soil bacterial communities.

**Figure 6:**
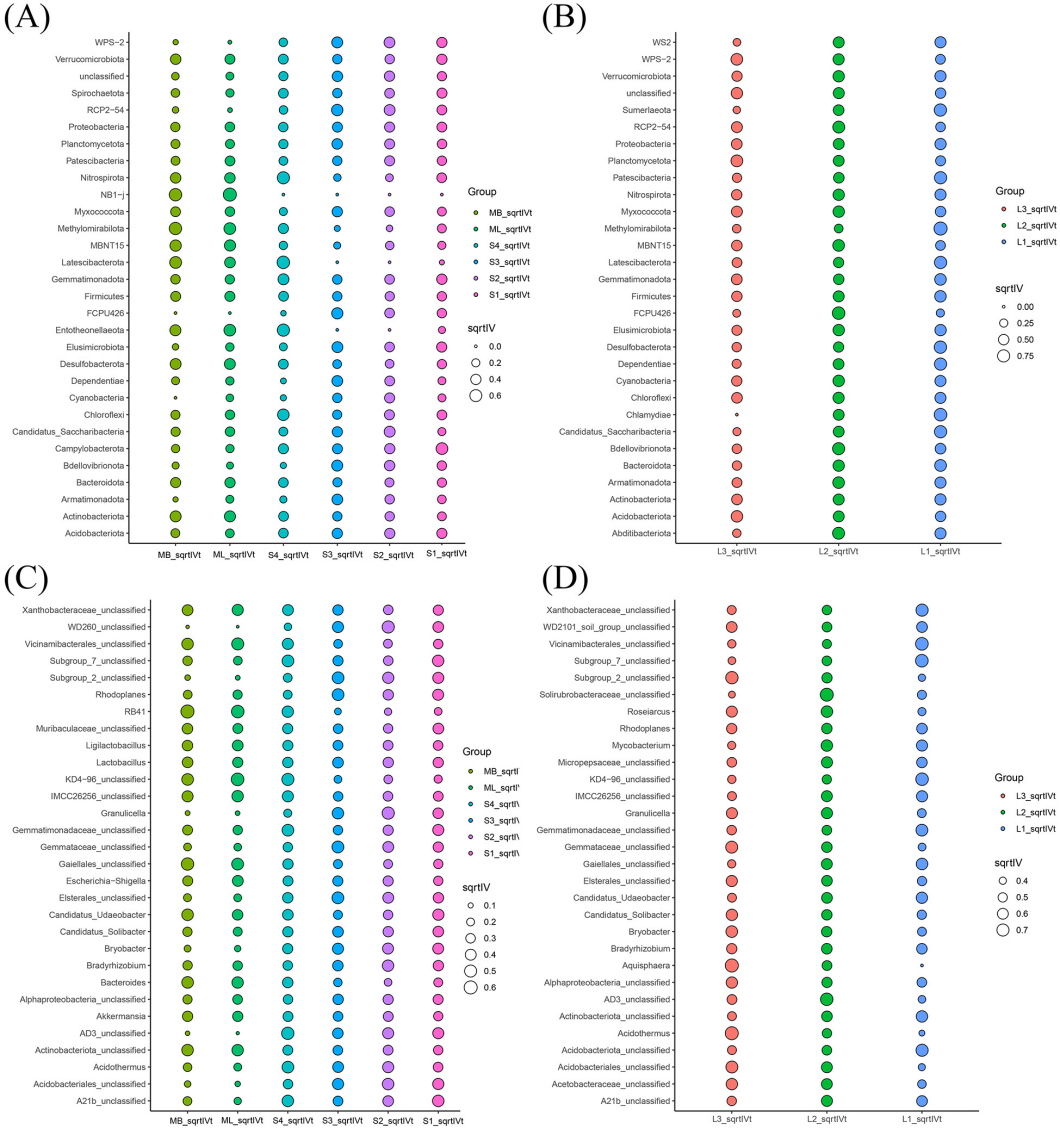
Soil bacterial indicator analysis bubble chart. **(A)** and **(B)** Size (circle size) of the sqrtIVt value at the genus level between the cutting and original forest plots. **(C)** and **(D)** Magnitude of the sqrtIVt values at the genus level between the cutting and original forest plots. The sqrtIVt value was the square root result of the indicator value. The larger this value of a certain species was, the more capable this species was of serving as a biomarker for this group. Threshold for the square root of the indicator value (sqrtIVt).

Indicator analysis of the soil bacterial phyla revealed significant differences in biomarkers among various plots: Campylobacterota (sqrtIVt value = 0.57) in S1, Candidatus_Saccharibacteria (0.47) in S2, RCP2-54 (0.53) in S3, Latescibacterota (0.67) in S4, NB1-j (0.75, 0.66) in ML and MB, respectively, Methylomirabilota (0.91) and Chlamydiae (0.83) in L1, FCPU426 (0.81) in L2, and Planctomycetota (0.69) in L3 (Figures 6A, B).

Indicator analysis of soil bacterial genera revealed significant differences in biomarkers among various plots: A21b_unclassified (sqrtIVt value = 0.46) in S1, *Granulicella* (0.51) and WD260 unclassified (0.51) in S2, Gemmataceae_unclassified (0.49) in S3, AD3 unclassified (0.53) in S4, KD4-96 unclassified (0.56) in ML, RB41 (0.60) in MB, Vicinamibacterales_unclassified (0.75) in L1, Solirubrobacteraceae unclassified (0.78) in L2, and Aquisphaera (0.79) in L3 (Figures 6C, D).

### 3.5 Soil bacterial function prediction

Based on the PICRUSt2 functional prediction results (which only represent preliminary inferences and require further validation using subsequent metagenomic or transcriptomic sequencing data), gene family annotations from the Clusters of Orthologous Genes (COG) database were used to compare functional composition across plots.

**Figure 7:**
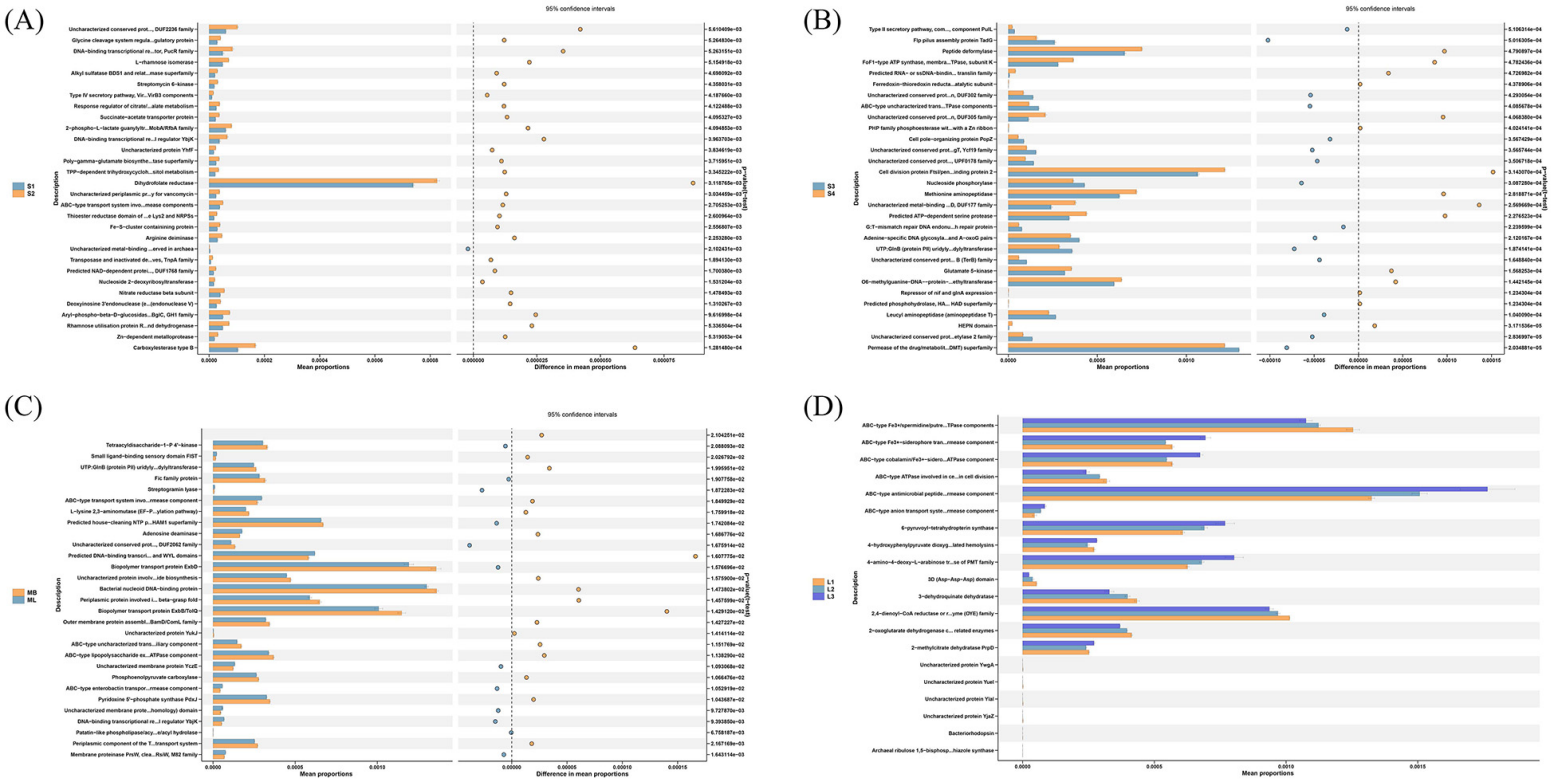
Soil bacterial functional prediction STAMP difference analysis map. **(A)**, **(B)**, **(C)**, and **(D)** Functional prediction STAMP differences of plots S1 and S2, S3 and S4, ML and MB, and L1, L2, and L3, respectively. The STAMP analysis in the figure above only presents the top 30 functions with p < 0.05 from pairwise t-tests (including 95% confidence intervals), allowing preliminary inference of associations between bacterial communities and these significant functions. The multiple group comparisons merely depict the abundance profiles, without performing any differential analysis. Statistical analysis of metagenomic profile (STAMP).

In the cutting plots, several significant differences in relative abundance were observed (Figures 7A–C): The activity of dihydrofolate reductase was significantly higher in S2 than in S1 (*p* < 0.01), which suggesting enhanced metabolic activity. The abundance of the drug/metabolite transporter (DMT) superfamily permease was significantly higher in S3 than in S4 (*p* < 0.001), which might suggest a heightened capacity for environmental information processing. The expression levels of cell division protein FtsI/penicillin-binding protein 2, peptide deformylase, methionine aminopeptidase, and O6-methylguanine-DNA–protein-cysteine methyltransferase (MGMT) were significantly higher in S4 than in S3 (*p* < 0.001), which suggesting enhanced functionality in genetic information processing. The predicted house-cleaning NTP pyrophosphatase and Maf/HAM1 superfamily were significantly more abundant in ML than in MB (*p* < 0.05), which suggesting their functional involvement in genetic information processing. The expression of biopolymer transport protein ExbD, bacterial nucleoid DNA-binding protein, biopolymer transport protein ExbB/TolQ, and a periplasmic protein involved in polysaccharide export, containing the SLBB domain of the beta-grasp fold, was significantly higher in MB than ML (*p* < 0.05), which may reflect enhancement in cellular processes.

In the original forest plots, the following differences were identified (Figure 7D): The ABC-type Fe^3+^/spermidine/putrescine transport systems, ATPase components, and 2,4-dienoyl-CoA reductase or related NADH-dependent reductase (Old Yellow Enzyme [OYE] family) were significantly more abundant in L1 than L2 and L3 (*p* < 0.05), which may underscore their potential roles in environmental information processing and metabolism. The ABC-type antimicrobial peptide transport system, permease component, 4-amino-4-deoxy-L-arabinose transferase or related glycosyltransferase of the PMT family, and 6-pyruvoyl-tetrahydropterin synthase were significantly higher in L3 than in L2 and L1 (*p* < 0.05). This observation may reflect enhanced functions in environmental information processing, cellular processes, and metabolism.

### 3.6 Correlation analysis of soil bacterial abundance and soil properties

**Figure 8:**
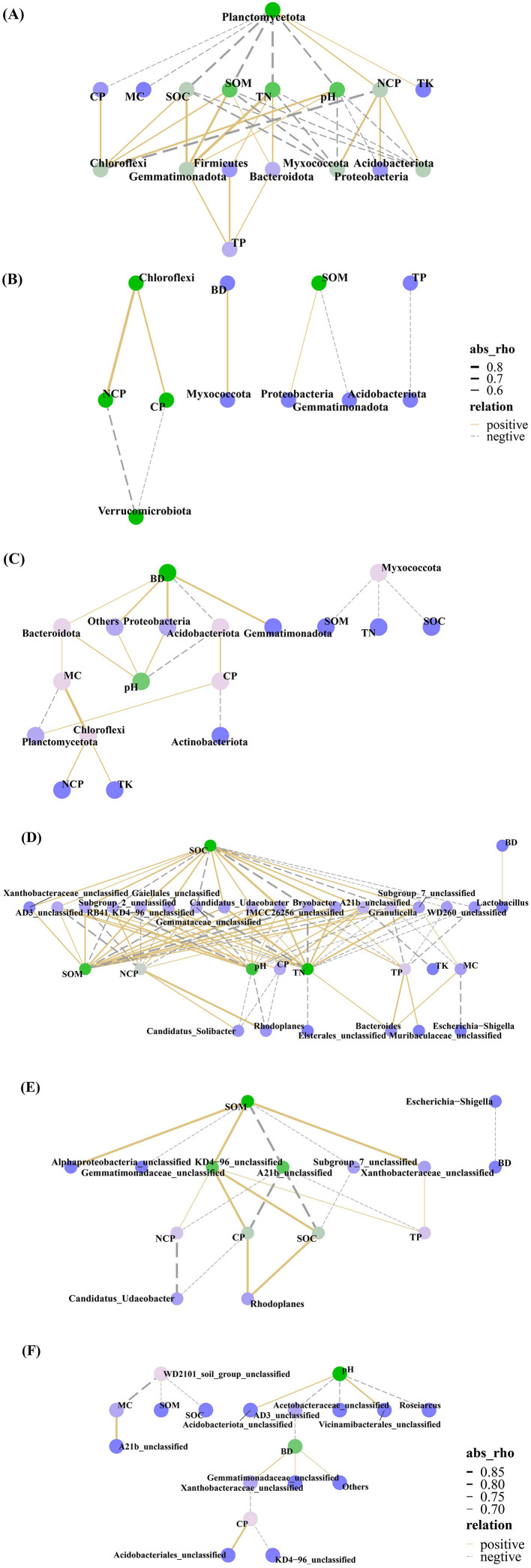
Correlation network of soil bacterial abundance and soil properties. **(A)**, **(B)** and **(C)** Correlation network maps of soil bacterial phylum abundance (top 10) and soil pH, SOM, SOC, TN, TP, TK, MC, BD, CP and NCP in the secondary gradual cutting, clear-cutting and original forest plots. **(D)**, **(E)** and **(F)** Correlation network heat maps for soil bacterial genus abundance (top 30) with the same soil properties for the secondary gradual cutting, clear-cutting and original forest plots. In the network diagram, the line thickness represents the strength of the correlation: the thicker the line, the stronger the correlation; the finer the line, the weaker the correlation. Solid lines indicate a positive correlation, while dotted lines point to a negative correlation. Soil organic matter (SOM), total nitrogen (TN), total phosphorus (TP), total potassium (TK), soil organic carbon (SOC), soil water (MC), bulk density (BD), capillary porosity (CP), and non-capillary porosity (NCP).

Correlations between the top 10 bacterial phyla, top 30 bacterial genera, and soil properties (pH, SOM, SOC, TN, TP, TK, MC, BD, CP, and NCP) were analyzed in both cutting and original forest plots, with significant relationships defined as rho > 0.5 and *p* < 0.05. A correlation network was subsequently constructed to visualize the relationships between bacterial abundance and soil physicochemical properties (Figure 8).

In the secondary gradual cutting plots (Figures 8A, D), soil properties were ranked by the number of their significant correlations with bacterial phyla and genera. For positive correlations, SOM had the highest count, followed by TN and SOC; then pH, TP, CP, and MC; and TK and BD had the lowest counts. For negative correlations, pH again exhibited the highest count, followed by TN; then SOC, SOM, NCP, TP, CP, and MC; and TK had the lowest count. Notably, several strong correlations were identified: TN was strongly positively correlated with Gemmatimonadota and Gemmatimonadaceae_unclassified (rho > 0.90) and strongly negatively correlated with Gemmataceae_unclassified (rho > 0.86). SOC and SOM exhibited a strong positive correlation with IMCC26256 unclassified (rho > 0.87) and a strong negative correlation with Bryobacter (rho > 0.83). pH showed a strong positive correlation with RB41 (rho = 0.85). NCP was strongly positively correlated with Rhodoplanes (rho = 0.85).

In the clear-cutting plots (Figures 8B, E), for positive correlations, SOM had the highest count, followed by SOC; then TN, pH, NCP, CP, and TP; and MC and BD had the lowest counts. For negative correlations, pH and TN again exhibited the highest counts, followed by SOC and NCP; then SOM, TP, CP, and MC; and TK had the lowest count. Notably, several strong correlations were identified: CP was strongly positively correlated with Chloroflexi (rho = 0.94) and strongly negatively correlated with Verrucomicrobiota (rho = 0.89). NCP was strongly positively correlated with Chloroflexi and Rhodoplanes (rho > 0.85) and strongly negatively correlated with Verrucomicrobiota (rho = 0.94). BD showed a strong positive correlation with Myxococcota (rho = 0.94). SOM was strongly positively correlated with Proteobacteria (rho = 0.89) and strongly negatively correlated with Gemmatimonadota (rho = 0.89). TP was strongly negatively correlated with Acidobacteriota (rho = 0.89).

In the original forest plot (Figures 8C, F), soil properties were ranked by the number of their significant correlations with bacterial phyla and genera. For positive correlations, BD had the highest count, followed by pH; then MC, CP, TK and NCP had fewer counts; SOM, SOC, TN and TP had no significant correlations. For negative correlations, pH exhibited the highest count, followed by CP; then SOM, SOC, MC, BD and TN showed fewer counts; TP, TK and NCP had no significant correlations. Notably, several strong correlations were identified: MC correlated positively with Chloroflexi and A21b unclassified (rho = 0.87) and negatively with WD2101 soil group unclassified (rho = 0.83). CP correlated positively with Acidobacteriales unclassified (rho = 0.83) and negatively with KD4-96_unclassified and Xanthobacteraceae unclassified (rho > 0.70). pH correlated positively with Vicinamibacterales unclassified and Acidobacteriota unclassified (rho > 0.75) and correlated negatively with AD3 unclassified, Roseiarcus, and Acetobacteraceae unclassified (rho = 0.73). BD correlated positively with Proteobacteria, Gemmatimonadota, Xanthobacteraceae unclassified, and Gemmatimonadaceae_unclassified (rho > 0.70) and correlated negatively with AD3 unclassified (rho = 0.72). SOM and SOC both correlated negatively with WD2101 soil group unclassified (rho > 0.70).

## 4 Discussion

Forest logging is an intense anthropogenic disturbance that systematically alters soil environments by modifying in-forest microclimates and litter inputs. This study demonstrates that different logging methods have very different effects on soil physicochemical properties compared to stable original forests. The capacity of original forest soils to maintain lower pH levels and stable nutrient cycling relies on their intact canopy structure and long-term ecological equilibrium. The dense canopy blocks direct sunlight from reaching the forest floor and thus slows mineralisation processes. Simultaneously, continuous and diverse litter inputs supply organic acids and sustain the SOM buffering system, while microbial decomposition keeps internal nutrient cycling efficient through microbially driven decomposition processes. The relatively low soil nutrient concentrations and reduced physical properties observed in original forest plots (L1–L3) represent inherent characteristics of late-successional stages, resulting from conservative nutrient cycling and long-term immobilization in climax communities, instead of ecosystem degradation or disturbance-induced declines. These mechanisms form the foundation for self-sustaining soil fertility in original forests (Fujii, 2014; Mou et al., 2024). Therefore, these stands serve as a valid reference ecosystem for assessing logging disturbance effects. In this context, clear cutting plot (ML), as a high-intensity disturbance, exerted long-term legacy effects that gradually shifted this equilibrium via decades of natural succession. Soil pH was much higher than in plots S1, S2, and L2 (*p* < 0.01). This phenomenon likely results from the synergistic effects of two mechanisms: a sharp decline in organic acid inputs and enhanced mineralisation rates. Canopy removal eliminates litter sources, reducing the SOM buffering capacity, while direct soil exposure to sunlight increases SOM mineralisation. This process releases substantial alkaline cations and raises soil pH (Liang, 2024). Conversely, secondary gradual cutting (S1) shows the benefits of moderate disturbance. The preserved canopy structure effectively moderates the microclimate through shading, demonstrating vegetation's crucial role in moisture retention, surface temperature reduction, and evaporation suppression. Consequently, MC remains substantially higher than in plot ML (Bereczki et al., 2024; Zhang et al., 2024; Liang et al., 2022). Notably, secondary gradual cutting and replanting of plot S4 showed even stronger recovery. SOM content was significantly higher than in unreplanted secondary gradual cutting plots S1 and S2 (*p* < 0.05). This observation indicates that replanting initiatives adds fine roots and litter, increasing carbon sources and stimulating soil microbial activity. This process facilitates the reconstruction of a vigorous root–soil interface similar to that in original forests during post-disturbance succession, thereby accelerating SOM accumulation and nutrient retention (Wang C. et al., 2024; Robinson et al., 2024). In contrast, high-intensity logging imposes persistent legacy effects on the long-evolved ecological balance of original forests, inducing soil nutrient alterations, while disturbance-mimicking logging approaches combined with active replanting interventions accelerate ecosystem recovery and succession (Zhou et al., 2022).

This study identified Proteobacteria, Acidobacteria, and Actinobacteria as the dominant bacterial phyla across all sampling sites, aligning with observations from various other forest ecosystems (Gao et al., 2019). Distinct logging regimes significantly altered the structure and function of bacterial communities. Furthermore, these communities were shaped by both long-term disturbance legacies and post-disturbance natural succession. In secondary gradual cutting plots (S1, S2), copiotrophic Proteobacteria and Actinobacteria were more abundant, a pattern closely linked to the resource pulse following moderate-intensity disturbance. These bacterial taxa help early-stage nutrient cycling by secreting extracellular enzymes that rapidly break down labile carbon sources (Wang et al., 2021). In contrast, the clear-cutting plot (ML) was dominated by oligotrophic Acidobacteria, which survive better under nutrient-limited conditions through enhanced carbon use efficiency (Li et al., 2019). Notably, secondary gradual cutting and replanting plots (S3, S4) showed significant enrichment of Chloroflexi and Firmicutes, likely because seedling roots released exudates that stimulated cellulolytic bacterial activity (Wang et al., 2021).

Soil bacterial diversity was lower in secondary gradual cutting plots than in natural birch forests but recovered to near-natural levels in plots subject to secondary gradual cutting and replanting. This observation suggests that moderate organic matter input increases soil bacterial diversity, corroborating findings by Yang and colleagues in the Daxing 'an Mountains (Yang et al., 2020). Additionally, original forest site L1 had the highest bacterial alpha diversity, attributable to a stable microclimate, abundant nutrient availability, and heterogeneous ecological niches. Conversely, L3 showed the lowest diversity, likely because key nutrient limitations or soil pH favored stress-tolerant microbial lineages and reduced overall diversity (Blaško et al., 2015; Panico et al., 2025; Lin et al., 2024). A noteworthy similarity in bacterial composition was observed among plots S4, ML, and MB, possibly due to common resource limitations or environmental filtering during the early stages of post-disturbance succession. This pattern aligns with the consistent recovery periods observed across all logged plots. Functional prediction analyses further revealed metabolic specialization among sites. For example, S2 was enriched in general metabolic functions, which is consistent with rapid resource exploitation post-disturbance. S3 had heightened environmental information processing capacity, suggesting acute environmental sensing and response. S4 and ML shared genetic information processing capabilities, implying convergent adaptive strategies. This convergence likely stems from enhanced gene regulation under similar environmental pressures—a potential adaptive response to disturbance legacies (Langille et al., 2013; Vaila et al., 2025). A limitation of this study is the low and unbalanced number of replicate plots, which may reduce the statistical robustness of beta diversity and indicator species analyses. Although all plots were independent true replicates, caution is warranted when extrapolating these findings to other forest regions.

Soil physicochemical properties function as important environmental filters that collectively shape the structure and function of forest soil bacterial communities. In this study, we identified soil pH, TN, SOM, and MC as the four predominant factors governing bacterial community assembly. Among them, soil pH exerted the strongest effect on bacterial community composition. Previous research has established that neutral to slightly alkaline soils maximize nutrient availability and provide optimal habitats for copiotrophic bacteria (Li et al., 2021; Rousk et al., 2009). In our study sites, soils were weakly acidic, and pH showed significant correlations with numerous bacterial phyla and genera (*p* < 0.05). Specifically, the strongest positive correlation emerged with the Acidobacteria subgroup RB41 (rho = 0.91). This pattern aligns with the low-pH conditions typical of birch secondary forests, where Acidobacteria proliferate. Furthermore, the strong enrichment of RB41 in MB plots indicates its adaptation to acidic, nutrient-poor soils and supports its use as a bioindicator of soil carbon flux (Stone et al., 2021). These findings are consistent with observations by Dai et al. (2018), who found that Acidobacteria declined at pH> 6.5, while Actinobacteria and Proteobacteria abundances substantially increased. Aside from pH, SOM serves as the principal energy source for microbial metabolism. Its quantity and quality jointly determine bacterial trophic strategies (Wu et al., 2013). As SOM complexity increases (e.g., higher cellulose and lignin), oligotrophic bacteria such as Chloroflexi, which degrade recalcitrant compounds, become dominant. In contrast, copiotrophic bacteria such as Proteobacteria, which rely on labile carbon, decline due to competitive exclusion (Zeng et al., 2025). This trophic specialization creates complementary metabolic networks: Chloroflexi stabilize carbon pools by decomposing complex organic matter, whereas Proteobacteria drive rapid nutrient mineralisation following ecosystem disturbances. Within nitrogen cycling processes, the high abundance of Planctomycetota in original forest plots (L1-L3) implies potential anaerobic ammonium oxidation (anammox) activity. This finding offers novel insights into nitrogen transformation pathways in forest ecosystems (Wang et al., 2025).

Soil moisture also modulates bacterial distribution by regulating oxygen diffusion. Elevated moisture in the clear-cutting plot reduced oxygen penetration, which suppressed aerobic bacteria such as Proteobacteria and promoted facultative anaerobes such as Actinobacteria (Sun et al., 2018). This mechanism explains the predominance of Actinobacteria in the clear-cutting plot, while secondary gradual cutting plots contained more Proteobacteria.

## 5 Conclusions

This study demonstrates that distinct logging methods differentially altered soil properties and bacterial community structure. These changes were closely interconnected and primarily driven by long-term disturbance legacies and post-disturbance natural succession. The clear-cutting plots (ML and MB) had higher soil nutrient levels and bacterial diversity. The secondary gradual cutting plots (S1–S4) exhibited differential responses that depended on management intensity. Among them, plot S4 showed significant recovery in SOM and microbial activity. In contrast, the original forest plots (L1–L3) showed signs of ecosystem degradation. Three key findings emerge from this study. First, logging practices altered soil nutrient dynamics and physical structure. First, clear-cutting significantly enhanced SOM mineralisation and increased soil pH, whereas replanting promoted SOM accumulation—a process intrinsically linked to post-disturbance succession. Second, bacterial communities responded differentially to each logging treatment, with distinct biomarker taxa in each plot type and functional adaptations through metabolic specialization, and these adaptive characteristics were also affected by disturbance legacies. Third, strong correlations between environmental factors and microbial communities identified pH, TN, SOM, and MC as the main drivers of microbial community assembly. Together these findings provide a scientific basis for developing targeted forest management strategies that simultaneously optimize soil health and support essential microbial ecosystem functions.

## Data Availability

The original contributions presented in the study are publicly available. This data can be found here: https://www.ncbi.nlm.nih.gov/sra/PRJNA1445393.
